# Detection of Movement Related Cortical Potentials from EEG Using Constrained ICA for Brain-Computer Interface Applications

**DOI:** 10.3389/fnins.2017.00356

**Published:** 2017-06-30

**Authors:** Fatemeh Karimi, Jonathan Kofman, Natalie Mrachacz-Kersting, Dario Farina, Ning Jiang

**Affiliations:** ^1^Department of Systems Design Engineering, Faculty of Engineering, University of WaterlooWaterloo, ON, Canada; ^2^Department of Health Science and Technology, Aalborg UniversityAalborg, Denmark; ^3^Neurorehabilitation Engineering Department of Bioengineering, Imperial College LondonLondon, United Kingdom

**Keywords:** brain-computer interface (BCI), movement related cortical potential (MRCP), constrained independent component analysis (cICA), electroencephalogram (EEG), spatial filters

## Abstract

The movement related cortical potential (MRCP), a slow cortical potential from the scalp electroencephalogram (EEG), has been used in real-time brain-computer-interface (BCI) systems designed for neurorehabilitation. Detecting MPCPs in real time with high accuracy and low latency is essential in these applications. In this study, we propose a new MRCP detection method based on constrained independent component analysis (cICA). The method was tested for MRCP detection during executed and imagined ankle dorsiflexion of 24 healthy participants, and compared with four commonly used spatial filters for MRCP detection in an offline experiment. The effect of cICA and the compared spatial filters on the morphology of the extracted MRCP was evaluated by two indices quantifying the signal-to-noise ratio and variability of the extracted MRCP. The performance of the filters for detection was then directly compared for accuracy and latency. The latency obtained with cICA (−34 ± 29 ms motor execution (ME) and 28 ± 16 ms for motor imagery (MI) dataset) was significantly smaller than with all other spatial filters. Moreover, cICA resulted in greater true positive rates (87.11 ± 11.73 for ME and 86.66 ± 6.96 for MI dataset) and lower false positive rates (20.69 ± 13.68 for ME and 19.31 ± 12.60 for MI dataset) compared to the other methods. These results confirm the superiority of cICA in MRCP detection with respect to previously proposed EEG filtering approaches.

## Introduction

The movement-related cortical potential (MRCP) is a low frequency (0–5 Hz) negative shift in the electroencephalogram (EEG) signal, which has recently been used as an EEG modality for real-time brain computer interface (BCI) applications, particularly in neuromodulation systems (Mrachacz-Kersting et al., 2016). The ability to detect MRCPs with high accuracy and short latency (usually shorter than 300 ms) on a single trial basis is crucial for these applications. Specifically, the high demand on temporal precision has been shown to be fundamental in efficiently inducing plasticity in neurorehabilitation applications (Mrachacz-Kersting et al., 2012). Improvement in accuracy and latency of single-trial MRCP detection is therefore a relevant challenge. The amplitude of the MRCP is typically between 5 and 30 μV and therefore easily masked by other brain activities (Wright et al., 2011). Moreover, low frequency motion artifacts and the electrooculogram (EOG) have frequency bandwidths similar to the MRCP, but with much greater magnitudes. Thus, extracting a single trial MRCP from an EEG signal with high accuracy and minimal latency in real-time is a challenging task.

Spatial filtering is one of the most commonly used EEG signal processing approaches for artifact removal and improving the detection accuracy of cortical potentials. The MRCP has a well-defined spatial distribution, being located directly over the scalp area of the corresponding primary motor cortex region. For example, the MRCP accompanying an ankle dorsiflexion task is most pronounced over the apex (Cz of 10–20 montage). The most common spatial filters used in EEG-based BCI systems are the Common Spatial Pattern (CSP) (Blankertz et al., 2008), Laplacian spatial filter (LAP) (McFarland et al., 1997; Xu et al., 2014a,b), and Independent Component Analysis (ICA) (Bell and Sejnowski, 1995; Cardoso, 1999). CSP decomposes multi-channel EEG signals into distinct spatial patterns by solving a generalized eigenvalue problem. This method has been widely used to extract motor imagery-based BCIs, particularly in sensory-motor rhythm (SMR) (Ramoser et al., 2000; Blankertz et al., 2008) and has also been tested preliminarily in MRCP detection (Niazi et al., 2011). However, the performance of CSP is very sensitive to outliers, which are inevitable in real-time BCI applications (Blankertz et al., 2008). LAP calculates the second derivative of the instantaneous spatial voltage distribution for each electrode location, and thereby emphasizes the activity originating in radial sources immediately below the electrode (McFarland et al., 1997). LAP has been applied in MRCP detection (Xu et al., 2014a,b). ICA-based spatial filters have been also successfully used in a variety of EEG signal processing applications, such as artifact reduction and source localization (Xu et al., 2004; Jiang et al., 2015). However, there are limitations associated with the implementation of ICA, especially for real-time applications, as it requires manual selection of the desired components from the estimated sources.

The constrained ICA (cICA), also known as one-unit ICA (Zhang, 2008), is a recent approach introduced to overcome the manual intervention limitation of ICA. cICA is a spatial filter extended from ICA that uses a reference signal to automatically extract only the desired source, without requiring the manual selection procedure of traditional ICA-based methods. cICA has recently been applied for EEG signal processing applications (James and Gibson, 2003; Joshua and Rajapakse, 2005) and has been shown to be successful in extracting event-related cortical potentials (ERP), such as the P300 (Spyrou and Sanei, 2006; Lee et al., 2013), as well as removing ocular artifacts (Huang et al., 2011); however, cICA has not been used previously for the detection of MRCPs. In this paper, we present for the first time, the application of cICA for MRCP detection, including a systematic investigation of the efficacy of cICA in single-trial MRCP detection, and comparison of cICA performance with the previously proposed CSP, LAP, Infomax (Bell and Sejnowski, 1995), and JADE (Cardoso, 1999). The performance of these filters was evaluated both with metrics based on the morphology of the MRCP and on the detection accuracy. For quantifying detection accuracy, the filtered EEG was classified with the previously proposed Locality Preserved Projection (LPP) followed by Linear Discriminator Analysis (LDA) (Xu et al., 2014a).

## Materials and methods

### Data acquisition

#### Participants

The data used in the current study are part of the dataset previously reported in Jochumsen et al. (2015). In the following, the experimental protocol is briefly described for clarity. The full details of the experimental procedure can be found in Jochumsen et al. (2015). Twenty-four healthy participants (7 female and 17 male 27 ± 4 years old) without any prior BCI experience participated in the experiment. All procedures were approved by the local ethics committee (N-20130081), and the participants gave their written informed consent before the experiment.

#### Experimental procedures

The participants were seated in a chair, relaxed and with their foot fixed to a pedal. During the experimental session, the participants were instructed to perform ankle dorsiflexion following a visual cue display on a computer screen that was located at a distance of 1.5 m in front of them. The cue was presented with a custom-made program (Knud Larsen, SMI, Aalborg University) which provides the instructions by displaying *Ready, Focus*, and *Task* commands in 8–10 s intervals. The 24 participants were divided into two groups. The first 12 participants (Group 1) were asked to perform actual dorsiflexion (motor execution, ME), while the remaining 12 participants (Group 2) were asked to perform only motor imagery (MI) of the movement. Four contraction types were performed: fast contraction targeted at 20% maximum voluntary contraction (MVC), fast contraction targeted at 60%, slow contraction targeted at 20%, and slow contraction targeted at 60% MVC. In the visual cue, a moving cursor showed when and how fast the subject should perform the task. For each of the four contraction types, each participant performed approximately 50 trials of the ankle dorsiflexion task (ME or MI). The order of contraction types was randomized for both ME and MI sessions. The motor tasks were separated randomly between 8 to 10 s. For the purpose of this study, we only analyzed and report the results using the trials of fast 20% MVC, for both ME and MI tasks. For this particular task, the instruction shown on the screen for Ready, Focus, and Task commands lasted between 4–6, 3, and 1 s, respectively. The subjects focused for 3 s, followed by the execution phase 0.5 s to reach 20% MVC, and the contraction was maintained for 0.5 s, after which a rest period was given (between 4 to 6 s).

#### EEG recording

A multichannel EEG electrode system (32 Channel Quick-Cap, Neuroscan) and an EEG Amplifier (Numaps Express, Neuroscan) were used according to the international 10–20 system to obtain EEG signals. Ten electrodes placed at standard 10–20 positions FP1, F3, Fz, F4, C3, Cz, C4, P3, Pz, and P4 were used to collect EEG data at a sampling rate of 500 Hz. The reference electrode was located on the right ear lobe. All analyses presented below were performed offline.

### Data processing

Since zero-phase non-causal IIR filters have been shown to perform well on Slow Cortical Potentials (SCPs) related to anticipatory behavior (Garipelli et al., 2011), the EEG data in the current paper were non-causally bandpass filtered between 0.05 to 3 Hz using a zero-phase second-order Butterworth bandpass filter prior to further processing. The choice of the filter was consistent with prior studies that used MRCP for real-time detection of motor intentions (Xu et al., 2014a) and similar to the recommendations of (Garipelli et al., 2011). All data were analyzed without rejecting segments with artifacts.

#### cICA for MRCP detection

The cICA approach is briefly explained in the following.

Suppose that a *N*-dimensional observed sensor signal $x\left(t\right)={\left[x_{1}\left(t\right),x_{2}\left(t\right),\ldots ,x_{N}\left(t\right)\right]}^{T}$ can be expressed as:

(1) x(t)=As(t),

where $s\left(t\right)={\left[s_{1}\left(t\right),s_{2}\left(t\right),\ldots ,s_{M}\left(t\right)\right]}^{T}$ is a *M*-dimensional mutually-independent latent source vector, and *A* is an unknown non-singular mixing matrix. The objective of cICA is to find a separating or de-mixing vector *w* without knowing the source vector and mixing matrix, such that:

(2) $$y(t)=w^{T}x(t)=w^{T}As(t),$$

where *y*(*t*) is the desired independent component (desired source signal). To determine this de-mixing vector, the cICA algorithm consists of the following steps. First, a linear whitening transformation is applied to the time series so that each column of *z*(*t*) has unit variance and the columns are uncorrelated, i.e., the covariance matrix of *z*(*t*) becomes the identity matrix:

(3) z(t)=Vx(t),

where *V* is a whitening matrix (Zhang, 2008). Next, according to the negentropy maximum criterion (Hyvärinen et al., 2001), the objective function of the next step is defined by:

(4) $$J(y)\approx \gamma {\left[E\{G(y(t))\}-E\{G(\nu )\}\right]}^{2},$$

where *E*{} indicates expectation of the signal and *y*(*t*) = *w*^T^*z*(*t*) is the output of the algorithm, γ is a positive constant, ν is a Gaussian variable having zero mean and unit variance, and G (·) can be any non-quadratic function. For traditional ICA methods, which have several independent components at the output, all columns of the output will be independent of each other by maximizing (4). To obtain one specific source signal, *a priori* information about the particular desired source needs to be incorporated into the cost function. In order to achieve this goal, the cICA problem is formulated as:

(5) $$\begin{array}{l} \;J(w)\approx \gamma {\left[E\{G(w^{T}z)\}-E\{G(v)\}\right]}^{2}\\ \text{Subject to}:g(w)=\varepsilon (y,r)-\xi ,\;h(w)=E\{y^{2}\}-1=0, \end{array}$$

where ε(*y,r*) is the similarity measure between the independent component *y* and the reference signal *r*, and ξ is a the similarity threshold. Therefore, *g*(ω) is the similarity constraint for the ICA optimization criterion, and *h*(ω) constrains *y* to have unit variance. Assuming that the desired IC is the one and only one closest to the reference *r*, one can get the following inequality relationship:

(6) $$\varepsilon (w^{*T}z,r)<\varepsilon ({w_{1}}^{T}z,r)<\ldots \varepsilon ({w_{N-1}}^{T}z,r),$$

where the optimum vector ω^*^ is the optimum demixing vector corresponding to the desired IC, and *w*_*i*_(*i* = 1, …, *N* − 1) corresponds to other unwanted ICs. The value of the similarity threshold lies in [$\varepsilon \left({w^{*}}^{T}z,r\right),\varepsilon \left({w_{1}}^{T}z,r\right)$]. The Lagrange multipliers method is used to solve the optimization problem of (5) (Lu and Rajapakse, 2005, 2006; Zhang, 2008):

(7) $$\begin{array}{l} w_{t+1}=w_{t}-\eta R_{z}^{-1}\Gamma_{1}/\Gamma_{2}\\ \;\Gamma_{1}=\bar{\gamma }E\{zG_{y}^{\prime }(y)\}-1/2\mu E\{g_{y}^{\prime }(y)\}-\lambda E\{zy\}\\ \;\Gamma_{2}=\bar{\gamma }E\{zG_{y^{2}}^{\prime \prime }(y)\}-1/2\mu E\{g_{y^{2}}^{\prime \prime }(y)\}-\lambda , \end{array}$$

where *t* represents the iteration number. $R_{z}=E\left\{zz^{T}\right\}$, $\bar{\gamma }=\gamma \cdot sign\left(E\left\{G\left(y\right)\right\}-E\left\{G\left(v\right)\right\}\right)$; and $G_{y}^{\prime }\left(y\right),g_{y}^{\prime }\left(y\right)$, $G_{y^{2}}^{\prime \prime }\left(y\right),g_{y^{2}}^{\prime \prime }\left(y\right)$, are respectively, the first and second derivatives of *G*(*y*), *g*(*y*) with respect to *y*. The optimum multipliers μ and λ are found by iteratively updating them based on a gradient-ascent method:

(8) $$\begin{array}{l} \mu_{t}=\text{Max}\{0,\mu_{t-1}+\eta g(w_{t-1})\}\\ \lambda_{t}=\lambda_{t-1}+\gamma_{t-1}h(w_{t-1}) \end{array}$$

Designing the reference signal plays a crucial role in cICA. The reference signal should be closely related to the desired source signal in terms of shape and phase (Zhang and Zhang, 2006; Zhang, 2008). For example, it is possible to use one of the observed channels as a reference signal (Mi, 2014). We propose the use of the average MRCP from Cz (for dorsiflexion) over all trials of a training set to build a subject-specific reference signal. Details of the training sets and construction of the reference signal using the training sets are discussed below.

#### Movement detection analysis

“Go” epochs and “No-go” epochs were extracted from the recorded signals according to the onset of the performed dorsiflexion task. Go epochs were the time intervals containing the MRCP whereas No-go epochs contained only noise. The effect of the filters on the MRCP morphology was quantified by two indices: the Signal to Noise Ratio (SNR) and the Go epoch variability (ρ). Moreover, three additional indices were calculated from the dataset of each subject to evaluate the performance of spatial filters in MRCP detection: True Positive Rate (TPR), False Positive Rate (FPR), and Detection Latency (DL). This was done using an offline evaluation framework, as described next. Following the extraction of Go epochs and No-go epochs, cross validation was implemented, and in each fold of the cross-validation, two thirds of the Go epochs and No-go epochs were randomly selected as the training set, and the remaining third of the Go and No-go epochs formed the testing set. Cross validation was performed whereby two thirds of the trials from the entire data set were randomly selected as a training set and the remaining one third as the testing set, and this was repeated ten times. The training set was used to generate the weights for spatial filters, and by assuming that the characteristics of the MRCP signals did not change across sessions, the demixing vector obtained from the training phase was applied to the test data. This offline evaluation over a number of folds allows a systematic evaluation of each method's performance by obtaining the receiver operating characteristics (ROC) curve of each method through cross-validation.

The SNR was calculated for each subject by extracting Go and No-go epochs, respectively, from [−2, 2] s and [2, 6] s with respect to the task onset (the turning point of the cue, see (Jochumsen et al., 2015)). Denoting the *l*th Go epoch and No-go epoch by $x_{S}^{l}\left(t\right)$ and $x_{N}^{l}\left(t\right)$, respectively, each containing *T* samples, the SNR can be expressed as:

(9) $$SNR=\frac{\sum_{l\;=\;1}^{L}\sum_{t\;=\;0}^{T}{\left[x_{S}^{l}(t)\right]}^{2}}{\sum_{l\;=\;1}^{L}\sum_{t\;=\;0}^{T}{\left[x_{N}^{l}(t)\right]}^{2}}.$$

The Go epoch variability ρ was defined as:

(10) $$\rho =\frac{\frac{1}{LT}\sum_{l\;=\;1}^{L}\sum_{t\;=\;0}^{T}\left|x_{S}^{l}(t)-\bar{x_{S}(t)}\right|}{\text{max}\left[\bar{x_{S}(t)}\right]-\text{min}\left[\bar{x_{S}(t)}\right]},$$

where $\bar{x_{S}(t)}$ is the average of the *L* Go epochs. The lower the value of ρ, the more consistent the Go epochs are. It should be noted that the two indices are calculated for all spatial filter outputs.

TPR, FPR, and DL were calculated on Go and No-go epochs, respectively extracted from [−3, 1] s and [2, 6] s with respect to the task onset, for each subject. TPR and FPR for each fold of the testing set were defined as:

(11) $$TPR=\frac{\text{Total number of correctly detected Go epochs}}{\text{Total number of Go epochs}},$$

and

$$FPR=\frac{\text{Total number of incorrectly detected No-go epochs}}{\text{Total number of No-go epochs}}$$

The Go epoch interval used to calculate the measures of detection performance was chosen to be different from the Go-epochs used for SNR calculation because, considering the length of the moving window, the time interval [−2 2] s, which perfectly covers all MRCP components, cannot be used if one would expect negative detection latencies where detection happens before the movement execution (*t* = 0). It should be noted that since the time interval [−3, 1] covers most parts of MRCP, this choice does not affect the TPR values.

To train Infomax and JADE, the training sets were built by concatenating all Go epochs and all No-go epochs of the training set. This means that all concatenated Go epochs (randomly selected) formed the first half of the training set signals; and the corresponding second half of the training set signals was formed by the concatenation of randomly selected No-go epochs in each channel. This approach was chosen as it provided a consistent training process for each method, and furthermore, it enabled us to perform the cross validation process. A similar approach was used for cICA, with an additional reference signal for the EEG signals. The reference signal for cICA was constructed using two steps: first, a subject-specific MRCP template was generated by averaging all Go-epochs of the Cz epochs in the training set ([−2, 2] s with respect to the task onset). Next, considering that the training sets were concatenated Go and No-go epochs for the other methods (Infomax and JADE), the reference signal of cICA was built by repeating the MRCP template corresponding to the signal epochs and using zero for the No-go epochs. By knowing the actual occurrence time of the executed or imagined movements, this approach could be implementable in the training phase of an online application as well. To train CSP, No-Go epochs and Go epochs were provided to the algorithm in two different matrices built by placing Go epochs in the rows of the signal matrix and each No-Go epoch in the rows of the noise matrix. LAP is not a supervised method; therefore, no training was required.

A LPP-LDA classifier was used for classification of the Go and No-go epochs (Xu et al., 2014a). A sliding window with length 2 s and 50 ms shift was applied to each Go and No-go epoch. A detection occurred when *n* consecutive sliding windows resulted in detection at the output of the LPP-LDA classifier. The choice for *n* determines the sensitivity of the overall system. Therefore, by varying *n* from 1 to 10, the average (over subjects) ROC curve was derived through cross-validation on the testing dataset of all subjects. TPR is defined as the ratio of the number of correctly detected Go epochs to the total number of Go epochs in the testing set. Similarly, FPR is defined as the ratio of the number of false detections of No-go epochs to the total number of No-go epochs in the testing set. The detection latency is defined as the time difference between detection and movement onset for the executed movements, and between detection and task onset for the imagined movements, in each Go epoch.

### Statistical analysis

To investigate the effect of the spatial filtering method on SNR and ρ, Friedman's Two-way ANOVA was performed, where the factor was Methods with five levels (LAP, CSP, Infomax, JADE, and cICA). When a significant difference was observed, a multiple comparison (Bonferroni) was carried out to identify which methods were significantly different. The significance level of all tests was set at *p* < 0.05. Furthermore, in order to investigate the effect of the five methods on MRCP detection, two-way repeated measure ANOVA was performed on the ME and MI datasets, with fixed factor the spatial filtering algorithms (LAP, CSP, Infomax, JADE, and cICA) and random factor the subject (SUB, 12 levels). The main null hypothesis was that Methods was not a significant factor on TPR, FPR, and DL. When the null hypothesis was rejected, a multiple comparison (Tukey with Bonferroni correction) followed.

## Results

**Figure 1:**
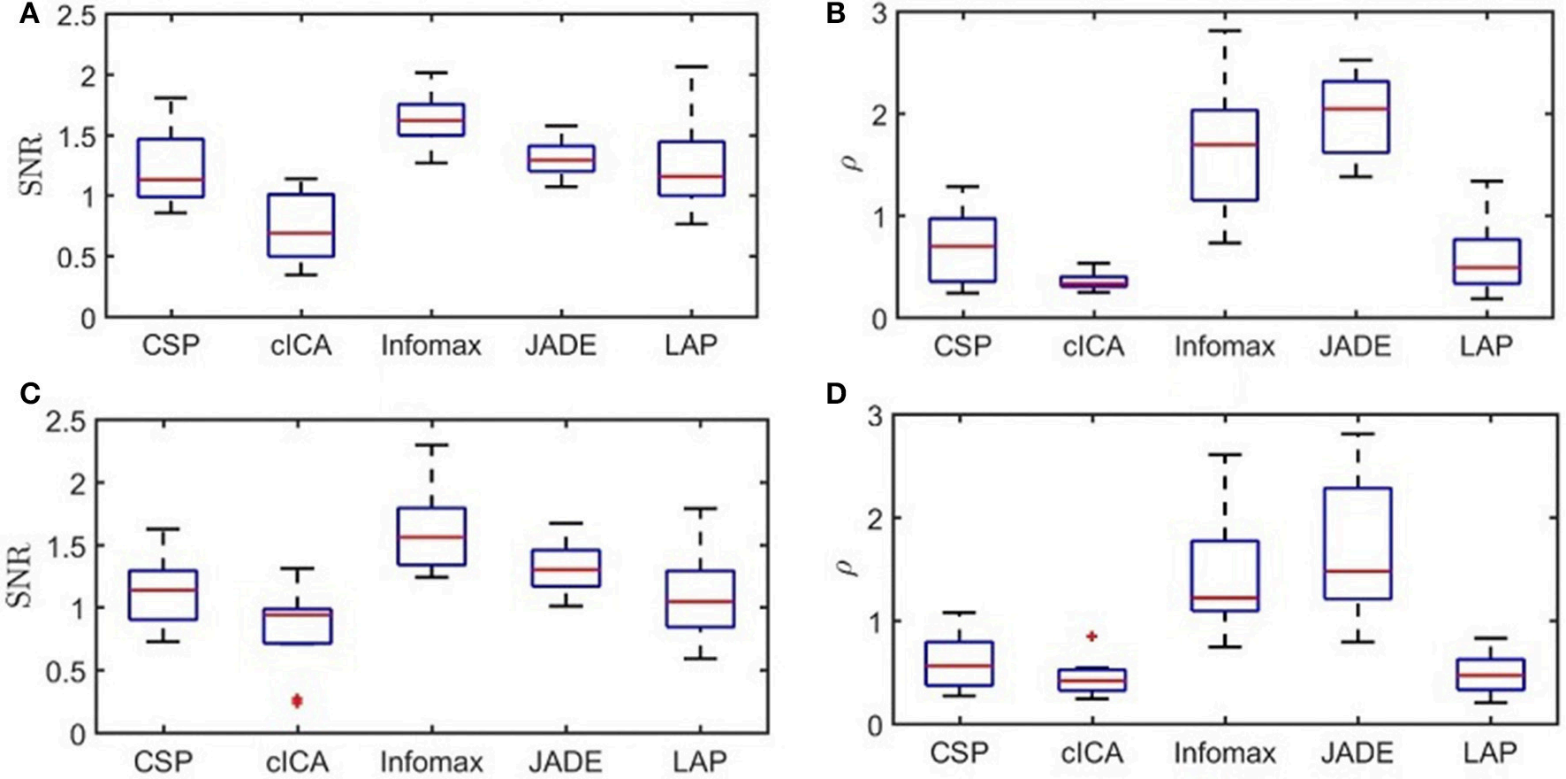
Boxplots of SNR and ρ-values for ME and MI datasets: **(A)** SNR values for ME dataset, **(B)** ρ-values for ME dataset, **(C)** SNR values for MI dataset, and **(D)** ρ-values for MI dataset.

The boxplot for the average values of SNR and ρ for the output of the spatial filters over folds from the testing sets, and for all subjects are presented in Figure 1. Direct observation indicates that, in this offline study, Infomax is able to suppress the noise better than other methods (highest SNR) in both the ME and MI datasets. In contrast, cICA had the lowest SNR values compared to other methods. However, in both the ME and MI datasets, cICA resulted in the lowest values for ρ among all methods. For the ME dataset, results from the Friedman's Two-way ANOVA showed that Methods had a significant effect on ρ and SNR (*p <* 0.001). The multiple comparison tests found that the SNR was smaller for cICA than LAP, Infomax, and JADE. Moreover, cICA, LAP, and CSP led to significantly lower variability compared to JADE and Infomax. For the MI dataset, the factor Methods again had a significant effect on ρ and SNR (*p <* 0.001). Post-hoc comparisons showed that SNR for cICA was significantly lower than Infomax, and JADE; and Infomax had significantly greater SNR values than LAP. For ρ, similar to the ME dataset, cICA, LAP, and CSP led to significantly lower variability than JADE and Infomax.

**Figure 2:**
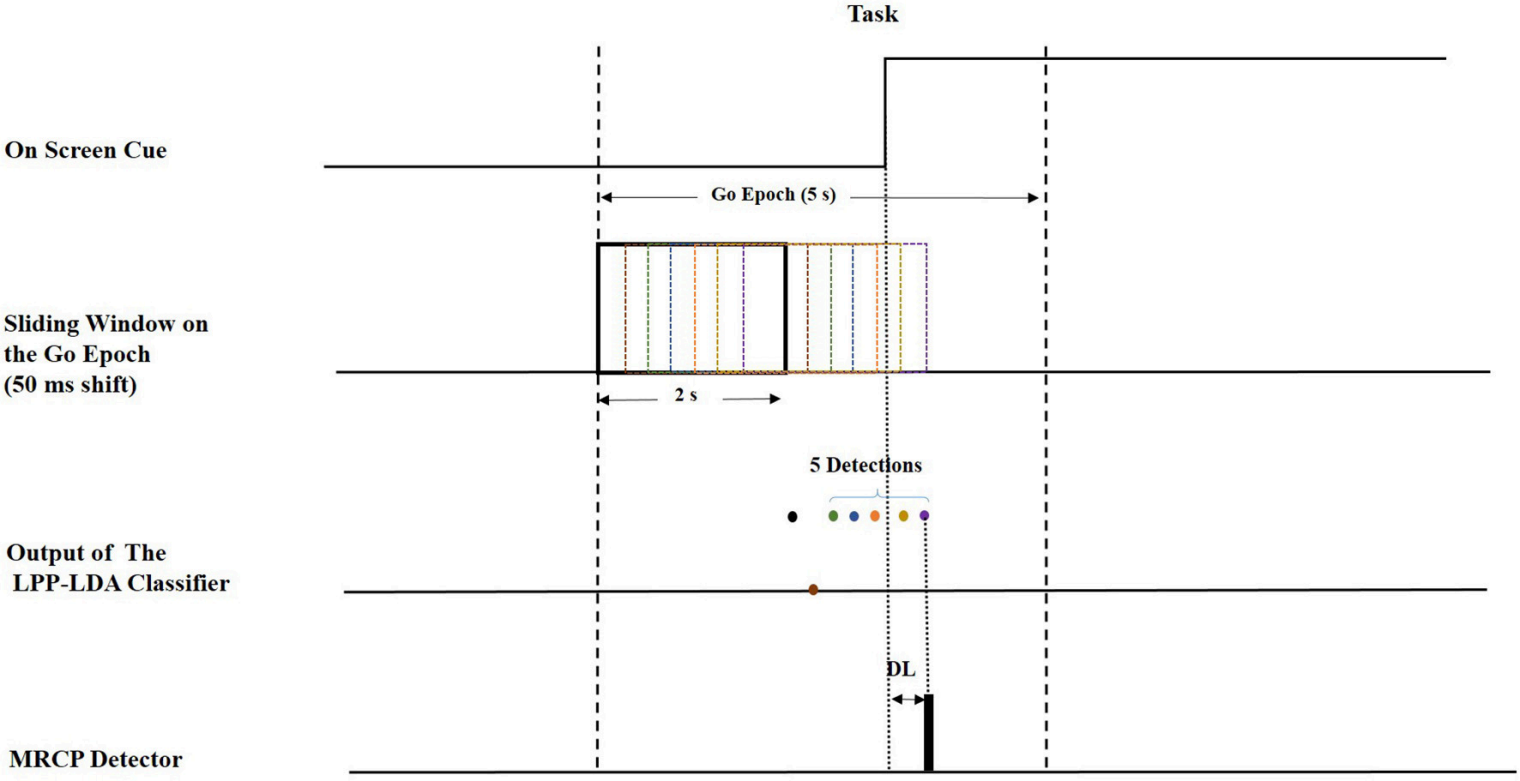
Offline implementation of movement detection.

**Figure 3:**
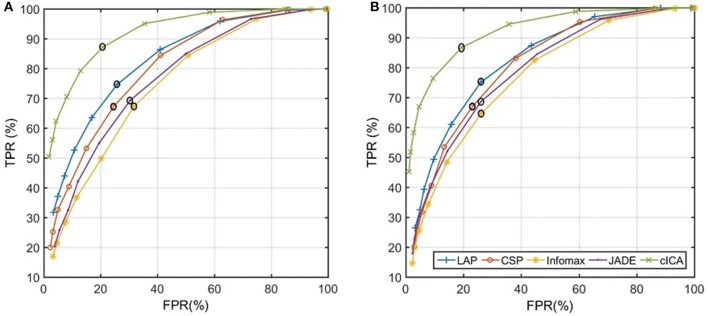
Average of the ROC curves of five spatial filters across all subjects: **(A)** ME dataset **(B)** MI dataset (black circle represents the value of each ROC curve when *n* = 5 in both graphs).

Figure 2 represents the algorithm used to calculate the detection latency when 5 consecutive windows result in detection at the output of the LPP-LDA classifier (*n* = 5). The average of the ROC curves of MRCP detection over all subjects for both ME and MI (testing) datasets is provided in Figure 3 for all spatial filters and 10 decision thresholds (*n* = 1, 2, …, 10). The area under the ROC curves is provided in Table 1. For both datasets, the area under the ROC curve of cICA has the highest value confirming that for each *n*, cICA provides the best combination of TPRs and FPRs (high TPR and low FPR). Therefore, the accuracy of cICA is superior compared to other spatial filters. As seen from the ROC curves, five decision windows are located at the midpoint of the convex part of the ROC curve, meaning that five consecutive detections could be a good balance between TPR and FPR for all filters. Therefore, the results presented next were calculated for five as the decision threshold.

**Table 1:** Average of the ROC curves of movement detection for ME and MI datasets.

**Spatial filter**	**Area under the ROC curve**				
	**LAP**	**CSP**	**Infomax**	**JADE**	**cICA**
ME dataset	0.81	0.79	0.73	0.75	0.90
MI dataset	0.80	0.79	0.76	0.78	0.91

The detection performance is presented in Table 2 for both ME and MI datasets. The highest TPRs and lowest FPRs and DLs were obtained for cICA for both datasets. The detection latency for cICA (−34 ± 29 ms for ME and 28 ± 16 ms for MI dataset) was significantly smaller than for the other spatial filters.

**Table 2:** Average TPR, FPR, and DL for movement detection for ME and MI datasets.

**Spatial filter**	**Motor execution**			**Motor imagery**		
	**TPR**	**FPR**	**DL (ms)**	**TPR**	**FPR**	**DL (ms)**
LAP	74.65 ± 13.13	25.83 ± 16.91	197 ± 15	75.06 ± 12.94	25.99 ± 17.04	216 ± 14
CSP	67.14 ± 13.99	24.55 ± 11.31	295 ± 13	66.87 ± 10.13	23.02 ± 10.56	246 ± 15
Infomax	67.27 ± 7.69	31.70 ± 9.94	245 ± 9	64.69 ± 9.42	26.19 ± 7.78	286 ± 11
JADE	69.33 ± 8.56	30.44 ± 10.26	256 ± 16	68.68 ± 10.35	26.12 ± 10.25	250 ± 13
cICA	87.11 ± 11.73	20.69 ± 13.68	−34 ± 29	86.66 ± 6.96	19.31 ± 12.60	28 ± 16

*The results are presented (mean ± standard deviation across subjects) for each spatial filter*.

For the ME dataset, the ANOVA test showed that Methods has a significant effect on TPR, FPR, and DL (*p* < 0.001). Multiple comparisons found that TPR for cICA (87.11 ± 11.73) was significantly higher than with all other methods. LAP (74.65 ± 13.13) had significantly greater TPRs than CSP (67.14 ± 13.99) and Infomax (67.27 ± 7.69). FPR for cICA (20.69 ± 13.68) was significantly lower than for Infomax (31.70 ± 9.94) and JADE (30.44 ± 10.26); and FPR for Infomax (31.70 ± 9.94) was significantly higher than for CSP (24.55 ± 11.31) and cICA (20.69 ± 13.68). Regarding the detection latencies, the statistical analysis showed that cICA (−34 ± 29 ms) had significantly lower detection latencies compared with all other methods. In contrast, the detection latencies with CSP (295 ± 13 ms) were significantly greater than for Infomax (245 ± 9 ms), LAP (197 ± 15 ms), and cICA (−34 ± 29 ms).

Results for the MI dataset were similar to those for the ME dataset. Methods influenced significantly TPR, FPR, and DL (*p* = 0.00 for TPR and DL, and *p* = 0.02 for FPR). Multiple comparisons indicated that TPR from cICA (86.66 ± 6.96) was significantly greater than for all other methods, and TPR for LAP (75.06 ± 12.94) was significantly higher than for CSP (66.87 ± 10.13) and Infomax (64.69 ± 9.42). FPR of cICA (19.31 ± 12.60) was significantly lower than for LAP (25.99 ± 17.04), Infomax (26.19 ± 7.78), and JADE (26.12 ± 10.25), but not significantly different from CSP (23.02 ± 10.56). The detection latency obtained with cICA (28 ± 16) was significantly lower than for all other methods.

**Figure 4:**
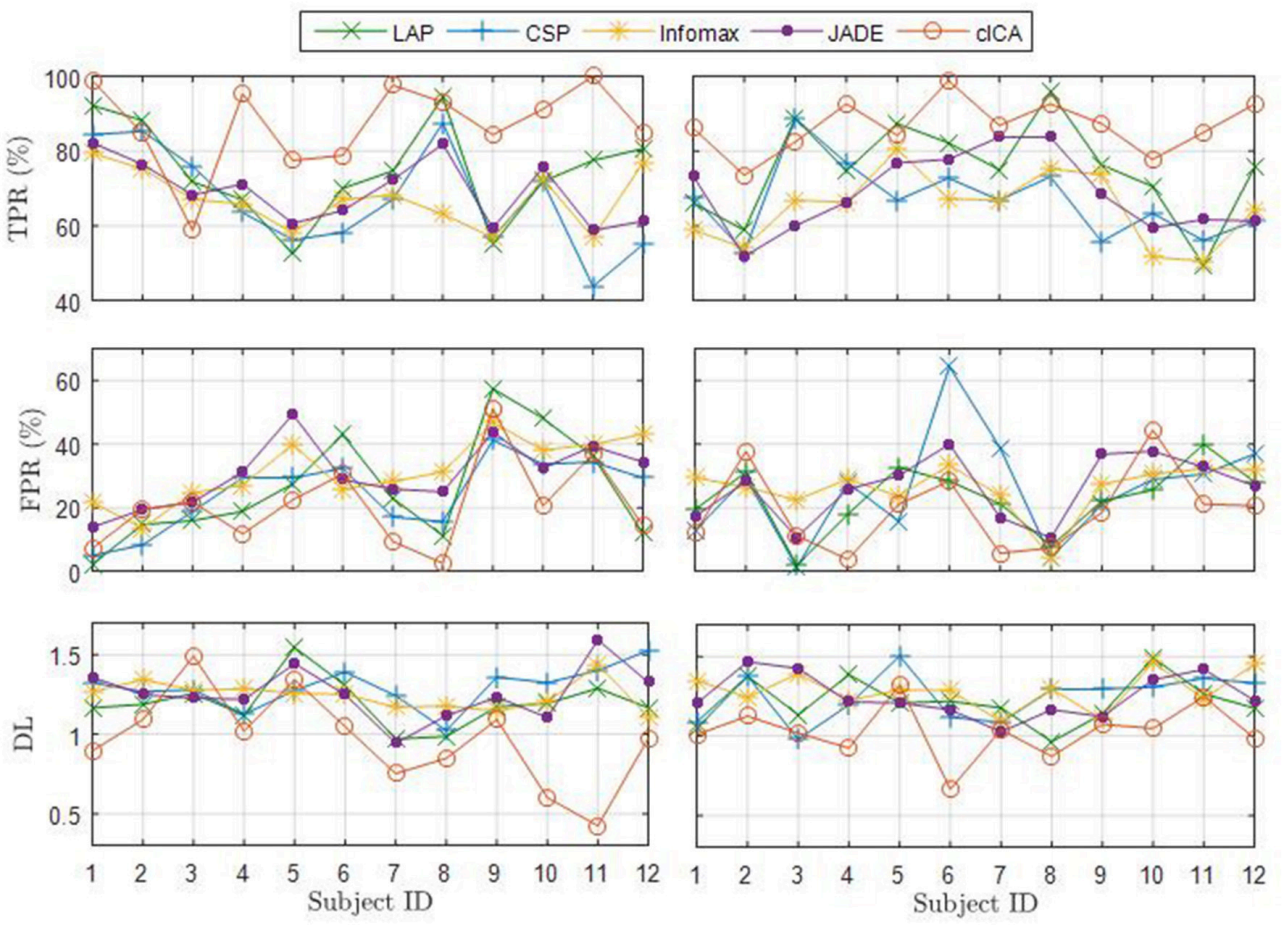
Average TPR, FRP, and DL for all subjects for both ME **(left)** and MI datasets **(right)**.

The average TPR, FPR, and DL over the 10 folds are reported for each subject from both datasets in Figure 4. For 11 of the 12 subjects, cICA has the highest TPR and lowest FPR and DL among all spatial filters.

## Discussion

The MRCP has recently been implemented as a control signal in a variety of BCI applications (Xu et al., 2014a,b; Jiang et al., 2015; Mrachacz-Kersting et al., 2016). The reliable and efficient detection of MRCPs enables the design of accurate and fast brain switches. Depending on the application of BCI systems, the importance of accuracy and latency of the system may vary. To be more specific, while large DL may not be ideal for BCI applications developed to induce brain plasticity, slightly lower TPR may not greatly affect the performance of the BCI system. On the other hand, high TPR are required for the control of exoskeletons for replacement rather than restoration of function, and for this application, a low DL is not so imperative. Accuracy and latency of detection of the MRCP highly relies on the signal processing method used to extract features from raw EEG. Spatial filters are one of the most efficient and successful feature extraction methods in EEG signal processing due to the spatial distribution of the signal features. In this study, the performance of cICA, a newly introduced ICA-based spatial filter, was compared with four other spatial filters in an offline experiment for MRCP detection from multi-channel EEG recordings, during execution and imaginary dorsiflexion of healthy subjects.

**Figure 5:**
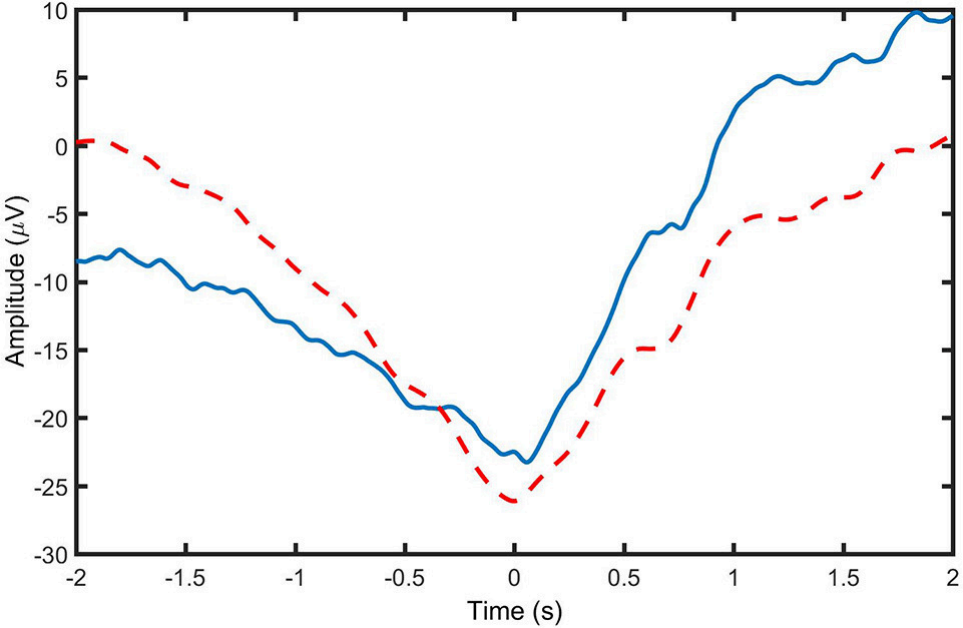
Average signal of all causally (___) and non-causally (- - - - -) filtered Go-epochs (MRCPs) from the Cz channel for Subject 1.

The performance of each spatial filtering algorithm in the detection of MRCPs was initially evaluated based on clarity and consistency of the extracted MRCP, quantified by SNR and ρ, respectively. Moreover, TPR, FPR, and DL were investigated through cross-validation in an offline experiment. The reported TPRs in this study are in agreement with the previous similar studies (Xu et al., 2014a,b). However, since, in this study, it was intended to evaluate the performance of the detector and determine the optimum parameters for movement detection using ROC, the values of FPR were calculated with a different measure than previous similar studies. In the previous studies, FPR was defined as the number of false detections per minute. Such approach for calculating FPRs caused the values of FPRs to be biased by the experiment protocol and inconsistent with TPRs. In this paper, the approach used to calculate FPR values makes the values independent of the experimental protocol, in which parameters such as the refractory period of the MI/ME can affect the accuracy of the definition of FPR used in previous studies (Niazi et al., 2011, 2013): false positive per unit time. Also, this approach is consistent with the approach used to calculate TPRs, enabling us to obtain ROC curves for the detector. The calculation of DL in this study is also in agreement with previous studies. It should be noted that a non-causal filter was used in the current study. In a real online experiment, a causal filter should be used. In order to investigate the effect of type of the bandpass filtering method (causal vs. non-causal), we performed an additional analysis to compare the performance of a causal second-order Butterworth bandpass filter with the bandwidth of 0.05–3 Hz with the same non-causal filter. The average signal of all causally and non-causally filtered Go-epochs (MRCPs) from the Cz channel for Subject 1 are provided in Figure 5. The observations indicate that there is a smaller amplitude in the negative peak of MRCP when the causal filter is used. We also compared the detection performance for causally and non-causally filtered signals for all subjects in the ME group. The causal filtering resulted in slightly higher FPR and lower TPRs compared to using non-causally filtered data, and the change was consistent in overall detection accuracy for all spatial filters investigated (the change of the averaged TPR values from causally to non-causally filtered signals was: 0.87, −0.19, −3.87, −5.89, −4; and the corresponding change of the averaged FPR values was: −7.69, 9.95, 7.46, 10.86, 13.47 for LAP, CSP, Infomax, JADE, and cICA respectively). This consistent change in overall detection accuracy is expected given the results shown in the figure, as the causal filter resulted in a less pronounced MRCP. However, causal filtering had no significant effect on the detection latencies (the difference between the averaged DL values for causally and non-causally filtered signals was: −0.07, −0.03, −0.06, −0.02, 0.00 s for LAP, CSP, Infomax, JADE, and cICA, respectively). Therefore, it is highly possible that the choice of causal or non-causal filtering has a slight effect on the overall detection accuracy, but there was no effect on DL values. However, this needs to be verified in a subsequent dedicated online study, which is beyond the scope of the current study with the objective of introducing cICA for MRCP detection.

The cICA requires the choice of a threshold that weights the relative importance of similarity with the reference signal in the optimization (Zhang, 2008). The suitable value of threshold depends on both the designed reference signal and the similarity measure. An effective way to determine the threshold given a reference signal, which was also used in this paper, is to use a small threshold initially, and then gradually increase the threshold (Lu and Rajapakse, 2006). For the reference signal based on the average of the Go epochs of the Cz channel, the value of the threshold was set to 0.9. On the other hand, as mentioned earlier, the shape of the designed reference signal plays an important role in the performance of cICA. Therefore, investigation of the effect of other types of reference signals such as the common rectangular pulse, smoothed MRCPs (Garipelli et al., 2013), and discriminative-based reference signal (Lee et al., 2016) will be done in the future in attempt to improve detection performance.

With the selected parameters, the area under the ROC curve for cICA was greater than for the other methods and cICA outperformed all the other filters for TPR and DL. Moreover, FPR was lower for cICA than for three of the other investigated methods. Overall, this indicates an improved performance of cICA with respect to previously proposed filtering methods. Considering that the detection of MRCP can be affected by hyper-parameters such as the overlap of the sliding windows and the number of detections required, further investigation will be done in the future to optimize the cICA algorithm based on these and other aspects. The averaged SNR values for the tested methods were not well associated with the detection performance. Indeed, cICA provided high TPRs and low FPRs compared to other methods but resulted in the lowest SNR values. One reason for the low SNR of cICA may be the optimization criteria of the method and the way SNR values were calculated in this study. The reference signal for cICA requires the algorithm to optimize the weights such that the desired signal can be obtained. As a result, the trial-by-trial consistency of the signal was improved by cICA. On the other hand, the results for the average ρ-values were more consistent with those obtained for TPRs. This is one of the findings of the current study: SNR does not necessarily correlate very well with detection performance, and the consistency of the Go epochs is equally (if not more) important for achieving a high detection performance. This likely stems from the fact that MRCP is a rather deterministic waveform, compared to other motor imagery BCI signal modalities, such as ERD/ERS. It can be concluded that, considering the shape of the reference signal applied in the current study, cICA seems to allow a more accurate modeling of the class of the Go epochs, and consequently a more pronounced effect on the sensitivity of the detector. This is because the choice of the reference signal can affect the ability of the cICA in modeling each class and separability of the classes. Therefore, cICA in the current study has limited effect on the specificity of the detector due to the choice of reference signal. It is possible that other types of reference signals can tune the algorithm to focus on other aspects of performance, such as specificity, which will be explored in future studies.

Regression analysis and template matching are also methods that have been used to extract desired EEG features and for EEG artifact removal (Wallstrom et al., 2004; Niazi et al., 2013; Urigüen and Garcia-Zapirain, 2015). Regression algorithms estimate the influence of the reference signal on the desired signal either in the frequency or time domain. Linear regression assumes that each EEG channel is the sum of the non-noisy source signal and a fraction of the source artifact that is available through a reference channel. Then, the goal of regression is to estimate the optimal value for the factor that represents such a fraction. Regression approaches need a reference channel to be able to operate automatically. In comparison, cICA is more flexible because although it uses a reference signal to extract features of the EEG signal or artifacts, the reference signal does not have to be a good estimation of the source(s). In fact, the reference signal can be very general, as long as it provides some reasonable constraint to ICA. For example, in Lee et al. (2016), a rectangular reference signal, which was not similar to the underlying source, was successfully implemented. In addition, since the regression methods are based on the time and frequency characteristics of the signals, they do not take into account the spatial information of the sources. Template matching techniques such as matched filter, which uses a template to maximize the SNR of the extracted signal, are also methods used for MRCP extraction (Niazi et al., 2013). Similar to regression, such methods only depend on the temporal features of the template and do not consider the spatial distribution of different sources. Also, matched filters are only optimal with additive Gaussian noise, so they are sensitive to other types of noise and artifacts.

In the current manuscript, we only used data from one of the four tasks for the purpose of introducing cICA for the first time in MRCP detection. Subsequent studies will be performed to investigate the generalizability of cICA when presented with data from different types of tasks.

## Conclusion

We have proposed a new spatial filter for MRCP detection. The proposed cICA extracts the desired signal by utilizing additional prior (spatial) information with respect to classic ICA, while exploiting higher order statistical structures as the CSP does. The results indicated that cICA did not enhance the extracted MRCP from multi-channel EEG significantly better than several commonly used spatial filters, including CSP, LAP, and ICA. However, cICA significantly outperformed these spatial filters in single-trial MRCP detection, with higher TPRs, lower FPRs, and shorter latency, both for ME and MI tasks. These results indicate that cICA is a promising new algorithm for detecting MRCP from multi-channel EEG. Following the promising results of the current study, we will conduct online experiments in a future study, in which cICA will be compared with LAP and CSP.

## Author contributions

Inception of ideas for the manuscript was by FK, NJ, JK, DF, and NM; conceptualization, methodology, validation, investigation, writing (review and editing) by FK, NJ, JK, DF, and NM; data acquisition/curation by NJ, DF, and NM; formal data analysis by FK and NJ; manuscript first draft by FK; manuscript revisions by FK, NJ, JK, NM, and DF; resources, supervision, administration, funding acquisition by NJ, JK, DF, and NM.

### Conflict of interest statement

The authors declare that the research was conducted in the absence of any commercial or financial relationships that could be construed as a potential conflict of interest.
